# In Vitro Screening of an In-House Library of Structurally Distinct Chemotypes Towards the Identification of Novel SARS-CoV-2 Inhibitors

**DOI:** 10.3390/ph17121668

**Published:** 2024-12-11

**Authors:** Michele Tonelli, Anna Sparatore, Ivan Bassanini, Valeria Francesconi, Fabio Sparatore, Kevin K. Maina, Serena Delbue, Sarah D’Alessandro, Silvia Parapini, Nicoletta Basilico

**Affiliations:** 1 Dipartimento di Farmacia, Università degli Studi di Genova, Viale Benedetto XV, 3, 16132 Genova, Italy; valeria.francesconi@edu.unige.it (V.F.); fabio.sparatore@emeriti.unige.it (F.S.); 2 Dipartimento di Scienze Farmaceutiche, Università degli Studi di Milano, Via Mangiagalli 25, 20133 Milano, Italy; 3 Istituto di Scienze e Tecnologie Chimiche “Giulio Natta”, Consiglio Nazionale delle Ricerche, Via Mario Bianco 9, 20131 Milano, Italy; ivan.bassanini@cnr.it; 4 Dipartimento di Scienze Biomediche Chirurgiche e Odontoiatriche, Università degli Studi di Milano, Via Pascal 36, 20133 Milano, Italy; kevin.maina@unimi.it (K.K.M.); serena.delbue@unimi.it (S.D.); nicoletta.basilico@unimi.it (N.B.); 5 Dipartimento di Scienze Farmacologiche e Biomolecolari, Università degli Studi di Milano, Via Balzaretti 9, 20133 Milano, Italy; sarah.dalessandro@unimi.it; 6 Dipartimento di Scienze Biomediche per la Salute, Università degli Studi di Milano, Via Mangiagalli 31, 20133 Milano, Italy; silvia.parapini@unimi.it

**Keywords:** SARS-CoV-2, COVID-19, riminophenazines, 4-aminoquinolines, 1,2,5-trisubstituted benzimidazoles, thiosemicarbazones, cytisine derivatives, arylamino enone derivatives

## Abstract

**Background/Objectives:** Four years after the COVID-19 pandemic, a very limited number of drugs has been marketed; thus, the search for new medications still represents a compelling need. In our previous work on antiviral, antiparasitic, and antiproliferative agents, we described several compounds (**1**–**13** and **16**–**20**) structurally related to clofazimine, chloroquine, and benzimidazole derivatives. Thus, we deemed it worthwhile to test them against the replication of SARS-CoV-2, together with a few other compounds (**14**, **15** and **21**–**25**), which showed some analogy to miscellaneous anti-coronavirus agents. **Methods**: Twenty-five structurally assorted compounds were evaluated in vitro for cytotoxicity against Vero E6 and for their ability to inhibit SARS-CoV-2 replication. **Results**: Several compounds (**2**, **3**, **10**, **11**, **13**–**15**, **18**–**20**) demonstrated antiviral activity (IC_50_ range 1.5–28 µM) and six of them exhibited an interesting selectivity index in the range 4.5–20. The chloroquine analogs **10** and **11** were more potent than the reference chloroquine itself and doubled its SI value (20 versus 11). Also, the benzimidazole ring emerged as a valuable scaffold, originating several compounds (**13**–**15** and **18**–**20**) endowed with anti-SARS-CoV-2 activity. Despite the modest activity, the cytisine and the arylamino enone derivatives **23** and **25**, respectively, also deserve further consideration as model compounds. **Conclusions**: The investigated chemotypes may represent valuable hit compounds, deserving further in-depth biological studies to define their mechanisms of action. The derived information will guide the subsequent chemical optimization towards the development of more efficient anti-SARS-CoV-2 agents.

## 1. Introduction

COVID-19 is an acute respiratory distress syndrome caused by SARS-CoV-2 (wild and mutated strains), which is an enveloped positive-sense, single-stranded RNA β-coronavirus. The disease may be fatal, and indeed, from the onset of the pandemic (November 2019) to the present day (autumn 2024), more than 770 million cases have occurred worldwide, with about 7 million deaths, according to WHO [1].

Many drug treatments have been attempted, but commonly with disappointing or, at the best, contradictory results [2,3]. In fact, even compounds that demonstrated very strong activity against the virus in in vitro assays have shown limited utility in clinical settings, and the reasons for their failure (ADME characteristics, rapid resistance onset, toxicity, etc.) are not fully elucidated [4,5].

**Figure 1:**
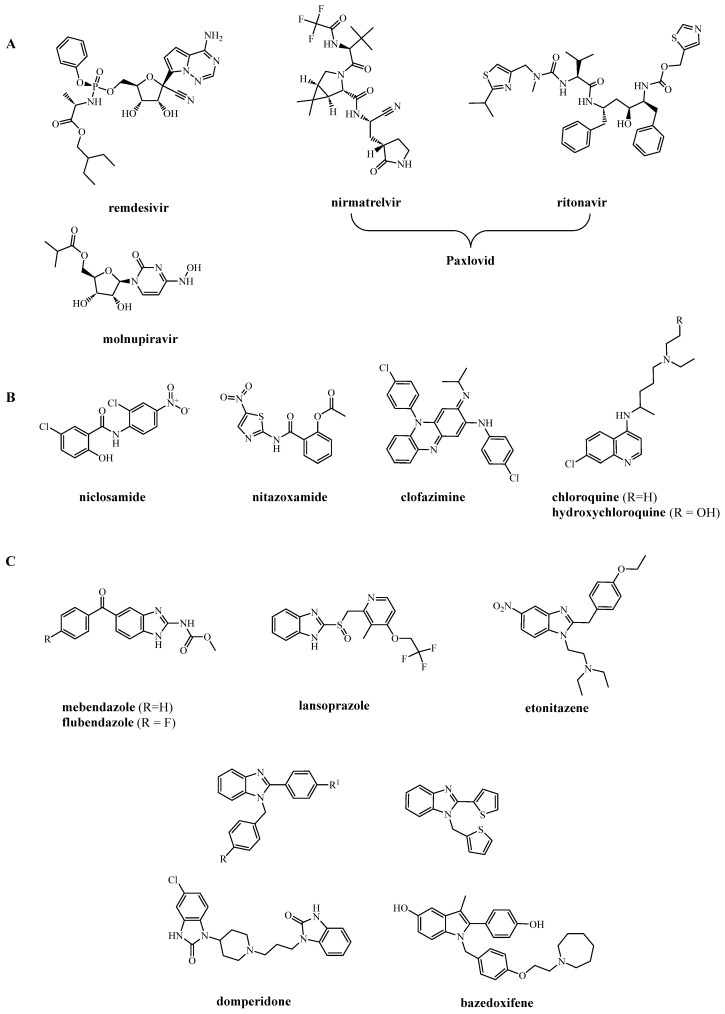
(**A**) American FDA- and European EMA-approved drugs for COVID-19 therapy; (**B**) largely used drugs even if not approved by regulatory agencies; (**C**) examples of benzimidazole (and similar rings) derivatives active against SARS-CoV-2.

At present, only a few known antiviral agents (structurally complex and rather expensive) have been approved for clinical use, even if with many limitations, such as remdesivir, Paxlovid (an association of nirmatrelvir and ritonavir), and molnupiravir (Figure 1A) [3]. Remdesivir was approved by the American FDA in 2020, although data regarding its ability to reduce mortality and length of hospitalization remain controversial [6,7,8]. Similarly, molnupiravir was approved in 2021, but in 2023 was declared to have no beneficial effects by EMA (24 February 2023) [9].

Besides the cited repurposed antiviral drugs (eventually associated with anti-inflammatory agents to alleviate symptoms and slow disease progression), a large number of natural and synthetic compounds have been tested against the virus and/or some of its functional components, leading to the definition of the most promising targets to be hit, such as SARS-CoV-2 main protease (Mpro), pre-fusion spike protein, and papain-like protease (PL) proteins [10,11,12].

**Figure 2:**
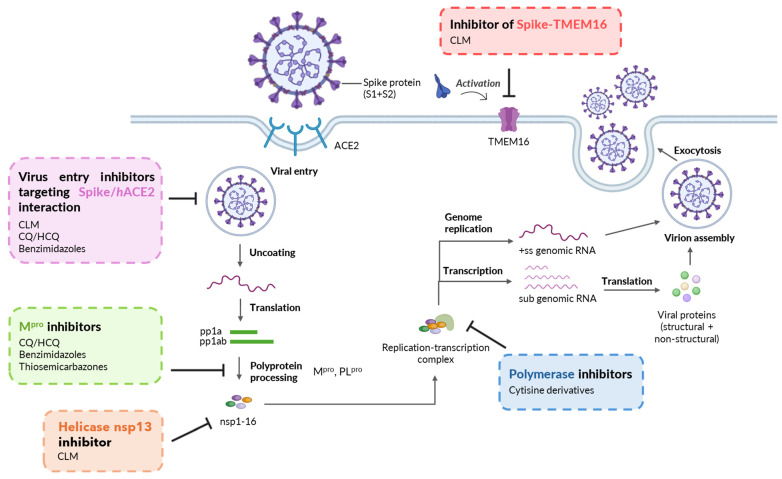
Mechanisms of SARS-CoV-2 inhibition showed by prototype drugs/molecules, which are structurally related to the chemotypes here investigated. Virus entry inhibitors: CLM [15], CQ/HCQ [19], Benzimidazoles [23,29]; Mpro inhibitors: CQ/HCQ [20], Benzimidazoles [27,28], Thiosemicarbazones [31,32]; Helicase inhibitor: CLM [15]; Polymerase inhibitors: Cytisine derivatives [33]; Inhibitor of Spike-TMEM16 activation: CLM [14]. Created with Biorender.com.

From a library of more than 3000 FDA-approved drugs, niclosamide, an old drug for tapeworm infection, emerged as the most potent inhibitor (IC_50_ = 0.28 µM) [13]. Niclosamide, along with clofazimine (an antibiotic used for the treatment of leprosy and drug-resistant tuberculosis), showed the most effective inhibition of spike-induced TMEM-16 activation (Figure 2), which leads to syncytia formation and thrombotic events [14,15]. Despite its antiviral potency, due to questionable bioavailability issues, niclosamide has currently been replaced by nitazoxanide (Figure 1B), an analogous salicylamide derivative. Recently, niclosamide has been reappraised as an anti-SARS-CoV-2 drug by restricting entry protein CD147 [16] and possessing effective anti-inflammatory activity in the respiratory tract of mice [17] at concentrations comparable to plasma concentrations attainable after oral administration.

During the early management of the pandemic, chloroquine (CQ) and hydroxychloroquine (well-known agents for treating malaria and rheumatoid arthritis) were largely used on the basis of an already known general and potent antiviral activity [18,19,20] (Figure 1B and Figure 2). Against SARS-CoV-2, they exhibited IC_50_ = 5.47 and 0.72 µM, respectively, but, unfortunately, these molecules provided limited to no therapeutic benefit in COVID-19 treatment because effective concentrations were not reached in vivo [21,22]. However, we feel it is still possible that some analogs of CQ may retain antiviral activity with ADME properties more appropriate for clinical use.

From an extensive screening of more than 12,000 compounds, examined by different physico-chemical and biological points of view, the benzimidazole nucleus resulted in a privileged structure for developing agents against SARS-CoV-2 [23,24,25,26] (Figure 2). Indeed, antimycotics such as mebendazole and flubendazole, proton pump inhibitors such as lansoprazole and analogs, analgesics such as etonitazene, and a miscellanea of 2-aryl-1-arylmethylbenzimidazoles (Figure 1C) exhibit potent activity against SARS-CoV-2 proteases [27,28] and spike proteins [23,29]. It is worth noting the high potency of the D2 antagonist domperidone (used as an antiemetic) and that of the estrogen receptor modulator bazedoxifene (used as an anti-osteoporotic). Although domperidone is a benzimidazolone [30] and bazedoxifene an indole [29] derivative, both possess a molecular geometry very close to that of benzimidazoles.

## 2. Results and Discussion

### 2.1. Design of the Study

**Figure 3:**
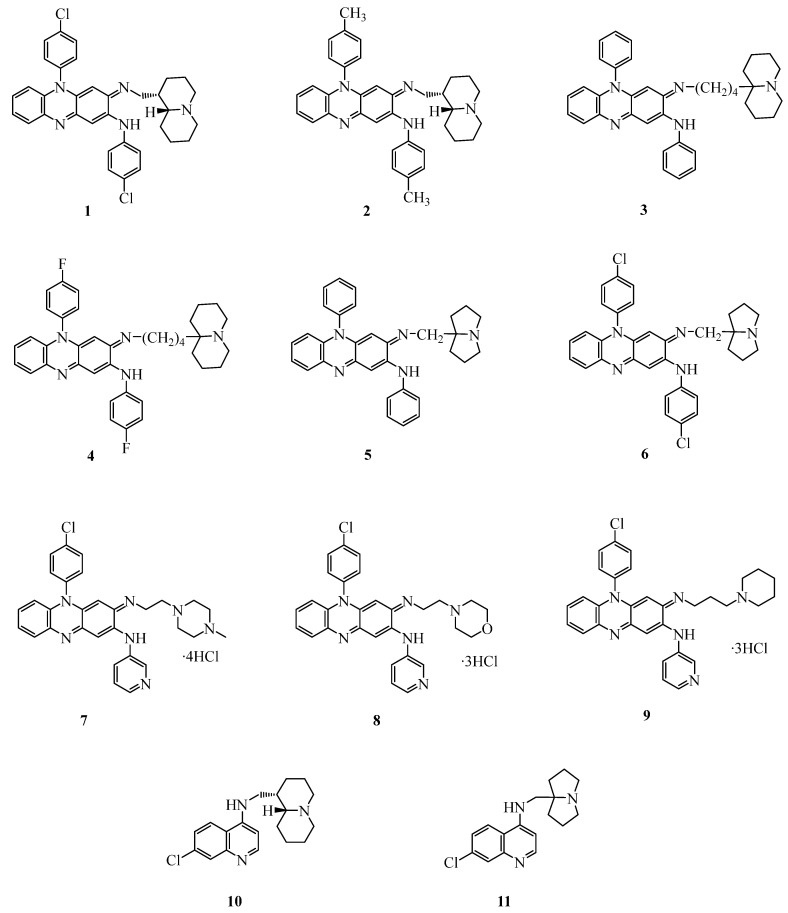
Structures of clofazimine and chloroquine analogs investigated as anti-SARS-CoV-2 agents.

**Figure 4:**
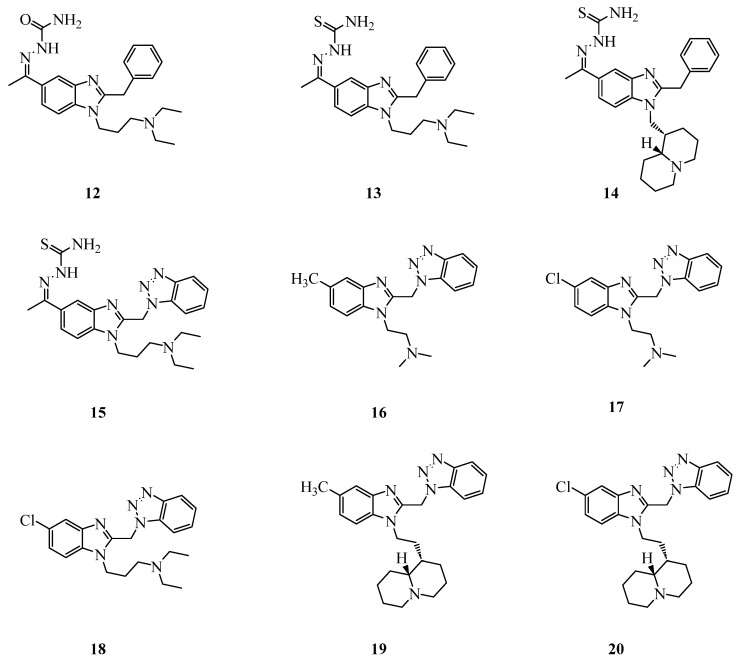
Structures of benzimidazole derivatives investigated as anti-SARS-CoV-2 agents.

**Figure 5:**
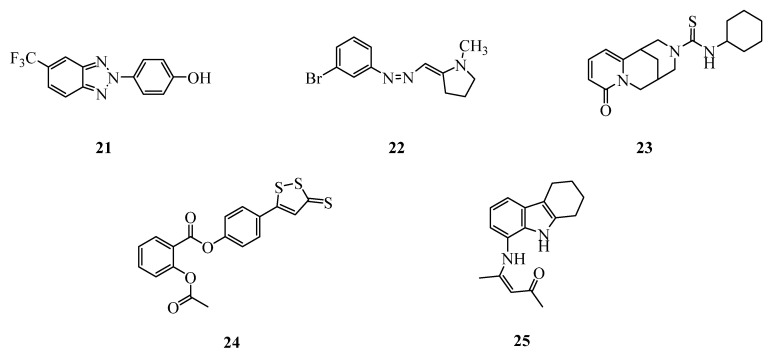
Structures of miscellaneous compounds investigated as anti-SARS-CoV-2 agents.

In our previous work on antiviral, antiparasitic, and antiproliferative agents, we described several compounds (**1**–**13** and **16**–**20**) structurally related to the above cited clofazimine, chloroquine, and benzimidazole derivatives. Thus, we deemed it worthwhile to test them against the replication of SARS-CoV-2, together with a few other compounds (**14**, **15** and **21**–**25**), which showed some analogy to miscellaneous anti-coronavirus agents (Figure 3, Figure 4 and Figure 5).

In the last ten years, through the introduction of basic moieties in the molecule of clofazimine (CFM), we obtained several CFM analogs with improved activity against *Mycobacteria*, *Plasmodia*, *Leishmania*, and other protozoa [34,35,36]. Among these compounds, a highly water soluble C3-aminopyridinylriminophenazine **7** (known as **MU17**) was found to also be endowed with improved anti-Wnt and anti-cancer activities (triple-negative breast cancer), and, most notably, to be devoid of drug-related skin coloration [37]. For these valuable characteristics, nine basic clofazimine analogs (**1**–**9**) (Figure 3), characterized by different degrees of lipophilicity, were first chosen for testing against SARS-CoV-2.

Through the exchange of the linear basic side chain of CQ with the cumbersome quinolizidinyl- and pyrrolizidinyl-alkyl moieties, we obtained potent anti-plasmodial agents active against CQ-resistant strains of *P. falciparum* and different species of *Leishmania* [38,39]. Two of these compounds (**10** and **11**, Figure 3) displayed favorable preclinical pharmacological profiles [40,41] and, hence, were selected for the present screening.

Among the many benzimidazole derivatives that we investigated as analgesics and antivirals, and somewhat related to the cited etonitazene (Figure 1C), compounds **12**–**20** (Figure 4) were selected as relevant to the present study. Compounds **12** and **13** exhibited moderate activity against influenza A H1N1 and coronavirus 229E [42], while compounds **16**–**20** [31,32,43] were active versus several viruses and showed a remarkable potency against RSV, interfering with the F protein-mediated fusion with the host cells [31]. Compounds **14** and **15** are new synthetized compounds that incorporate the thiosemicarbazone Schiff base with quinolizidine (**14**) or benzotriazole (**15**) motifs, which have previously shown to provide promising antiviral agents [31,32,43,44]. Thiosemicarbazone-based compounds have recently been demonstrated to inhibit SARS-CoV-2 by targeting Mpro [45,46] (Figure 2).

Finally, to further explore the chemical space important for developing novel anti-SARS-CoV-2 agents, we selected five compounds (**21**–**25**, Figure 5) from an in-house library, characterized by very different structures and biological profiles.

Compounds **21** and **22** have previously demonstrated a broad spectrum antiviral activity [44,47], including activity against Dengue virus type 2 (unpublished results, IC_50_ = 17 and 24 µM, respectively).

Compound **23** is a cytisine derivative that was studied as a nicotinic receptor ligand [48]; it is an analog of the 3-(N-allyl)cytisin thiocarbamide described by Russian authors as a very potent anti-dengue agent with EC_50_ = 0.1 µM [33]. The same authors have recently shown that other cytisine derivatives are able to inhibit SARS-CoV-2 RNA polymerase (RdRp) (Figure 2) [49].

The acetyl salicylic ester **24** (also known as MZe786) was studied by some of us as a gastric mucosa sparing aspirin analog [50]. The salicylic residue is also present in the cited nitazoxanide and niclosamide (Figure 1), potently active against SARS-CoV-2 [2,13].

Lastly, compound **25** (also known as FSAS-3), firstly prepared in 1968 by one of the authors [51], was subsequently found to possess anti-inflammatory activity. It is worth noting that compound **25** embodies in its structure the tetrahydrocarbazole moiety characteristic of the antiviral agent THC-19 [52], and the 3-phenylamino-2-propen-1-one residue of compound **6877002**, a well-known inhibitor of the interaction of CD40/TRAF6 [53] displaying anti-inflammatory and antitumoral activities [54]. Relations between TRAF6 inhibition and anti-SARS-CoV-2 activity are currently under investigation [55,56,57,58].

### 2.2. Chemistry

Most of the investigated compounds were re-synthesized as described in previous works: **1** and **2** [34], **3**–**6** [35], **7**–**9** [36], **10** [38], **11** [39], **12** and **13** [42], **16** and **19** [32], **17** and **18** [43], **20** [31], **21** [44], **22** [47], **23** [48], **24** [50], and **25** [51].

**Scheme 1:**
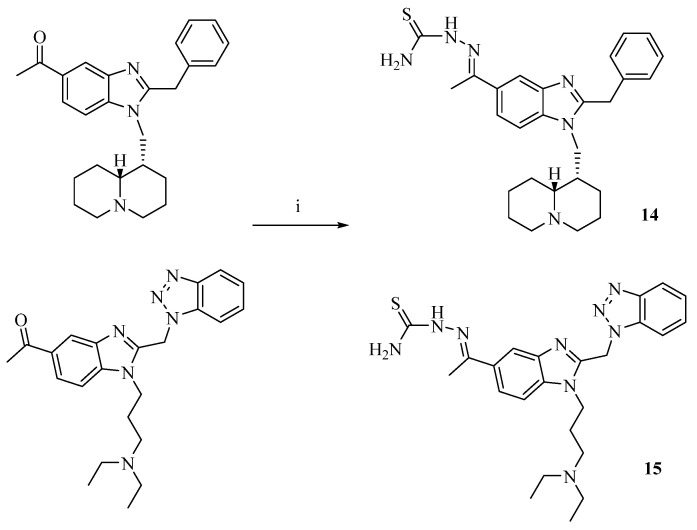
Reagents and conditions: (i) thiosemicarbazide in EtOH, H_2_O and glacial CH_3_COOH, 3 h at reflux.

The novel compounds **14** and **15** were prepared (Scheme 1) by reacting thiosemicarbazide with the relevant 5-acetyl benzimidazole previously described [43,59].

The structures of the novel compounds were confirmed using ^1^H and ^13^C NMR (see Supplementary Materials), and elemental analysis. The purity of compounds (checked by elemental analysis) was, in all cases, >95%. It is known that thiosemicarbazones can exist in two tautomeric forms due to thione-thiol tautomerism of the thioamide group. In the ^1^H NMR spectra of the Schiff bases **14** and **15**, the signal at 4.00 ppm attributed to the -SH proton is absent, thus suggesting that they adopt the thione form in DMSO [60]. Moreover, they display the secondary -NH- proton signal at 10.16/10.14 ppm, respectively; thus, it is reasonable that they are E isomers (range 9–12 ppm [60]). Finally, the C(S)NH_2_ protons appear as two signals, both because of the possibility of tautomerism which prevents the free rotation around the N-C bond, and because one of the protons can establish an intramolecular hydrogen bond with the sp2 nitrogen atom (forming a pseudo five-member ring), which would shift the corresponding signal to lower fields [61].

### 2.3. Biological Studies and SARS-CoV-2

Compounds **1**–**25** (Figure 3, Figure 4 and Figure 5) were evaluated in vitro for their antiviral activity against SARS-CoV-2 through real-time PCR, which measures the presence of the virus genome in the supernatants of control and treated infected cells. To determine non-cytotoxic doses suitable for use on SARS-CoV-2-infected cells, cytotoxicity against Vero E6 cells was assessed using the MTT assay. Non-toxic concentrations were then selected for antiviral screening against SARS-CoV-2. Results (IC_50_) are summarized in Table 1, which also includes cytotoxicity against Vero E6 cells (CC_50_) and the selectivity index (SI = CC_50_/IC_50_). Chloroquine was used as the reference drug.

**Figure 6:**
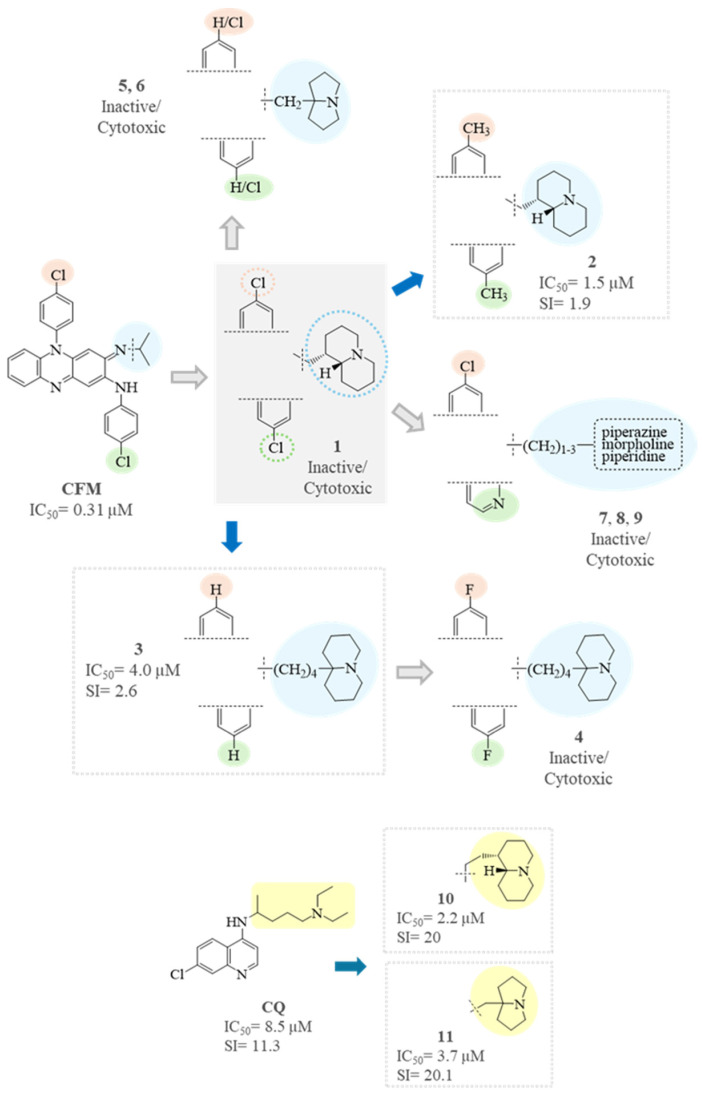
SAR analysis of CFM analogs (**1**–**9**) and CQ analogs (**10**–**11**).

In line with previous observations [34,35,36,37], the riminophenazine derivatives **1**–**9** exhibited relatively high cytotoxicity (CC_50_ = 1.74–10.5 µM), higher than that observed for all the other subsets of tested compounds (CC_50_ = 19–190 µM). On the other hand, different from the reference compound clofazimine (IC_50_ = 0.31 µM, [15]), these compounds exhibited only a low inhibition of virus replication with the best IC_50_ values of 1.5 and 4.0 µM for compounds **2** and **3** (SI = 1.9 and 2.6, respectively) (Figure 6). From the data presently available (particularly comparing compounds **3** and **7**), the molecular hydrophilicity, which is important to improve the anti-cancer and anti-Wnt activities of riminophenazines [37], seems to play an unfavorable role in anti-SARS-CoV-2 activity. It is worth noting that, in a parallel study [62], contrary to previous data, the inhibition of Wnt signaling by clofazimine and other inhibitors (like **7**, **MU-17**) did not result in a significant reduction in viral RNA load in the lung epithelium cell line used; clofazimine itself only achieved a 25% reduction in the tests. These issues surely deserve more in-depth studies.

Very interesting were the results for the chloroquine analogs **10** and **11**, which demonstrated dose-dependent activity and exhibited an SI (SI = 20) that was nearly double that of the reference drug CQ (SI = 11) (Figure 6). Both compounds have been previously studied for their in vitro and in vivo antimalarial activity and ADME-Tox profile [40,41], showing an excellent oral bioavailability with a high volume of distribution, good metabolic stability in microsomes and hepatocytes of different species, and low toxicity in mice (with a maximum tolerated dose, MTD, greater than 100 mg/Kg, i.p.), rats (oral MTD for **11** equal to 120 mg/Kg/day), and non-human primates (oral MTD for **11** equal to 50 mg/Kg/day). These results are in line with or even better than those obtained with chloroquine and are indicative of a good developability profile of the two compounds. Hence, the in-depth investigation of the activity of compounds **10** and **11** against SARS-CoV-2 is warranted, as is, on the other hand, the antiviral screening of other quinolizidine- and pyrrolizidine-derived chloroquine analogs synthetized in the past [38,39,63,64], which sometimes are endowed with even higher bulkiness and lipophilicity of the basic side chains.

**Figure 7:**
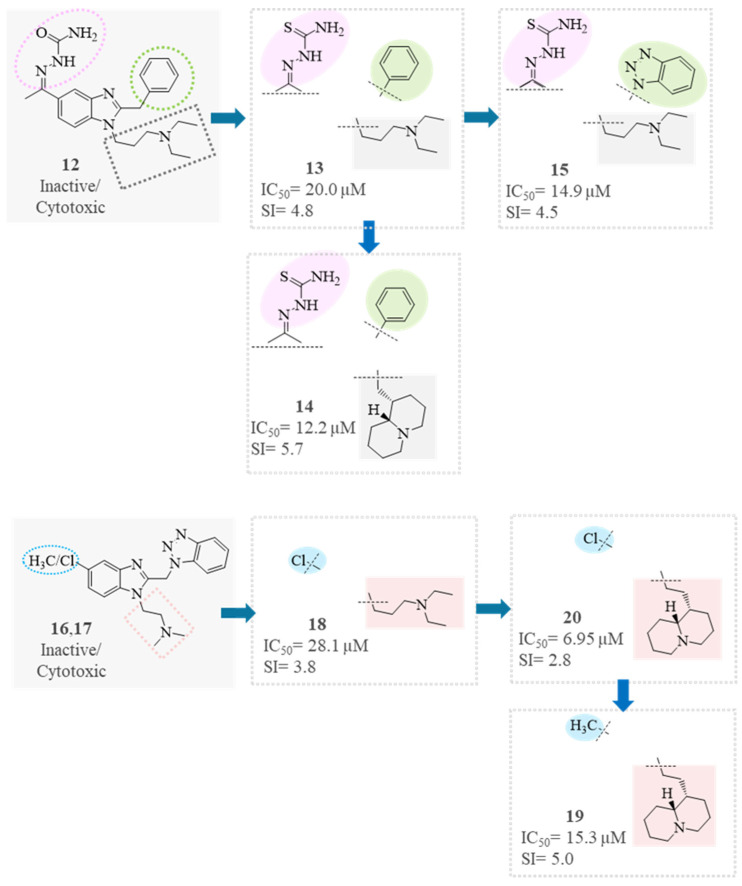
SAR analysis of benzimidazole derivatives (**12**–**20**).

In the subset of benzimidazole derivatives (**12**–**20**), six compounds (**13**–**15**, **18**–**20**) were moderately active and showed an SI in the range 2.8–5.7 (Figure 7). The net different activity observed for compounds **12** and **13**, despite the strict similarity of their structures, should mainly rely on the presence of the thiosemicarbazone group (Figure 7). Indeed, the thiosemicarbazone moiety and the embodied thiourea group are well-known antiviral pharmacophores, with studies addressing the activity versus different viruses [33,42,49,65,66,67], including SARS-CoV-2 [45,46,68], in relation to the molecular scaffold to which they are linked. Therefore, other thiocarbamido derivatives (analogs of **13**–**15** and **23**) are worthy of investigation.

In compounds **17**, **18**, and **20**, which differ in the increasing length of the basic side chain, both antiviral activity and toxicity increased accordingly (Figure 7). The exchange of the chlorine atom in position 5 (**20**) with a methyl group (**19**) reduced the activity but, even more, the toxicity; thus, compound **19** was found to be a better antiviral agent, with SI = 5 (Figure 7). On the contrary, the same exchange of groups for compounds **17** and **16** led to a strong increase in toxicity (CC_50_ = 191.5 µM → 37.7 µM).

Regarding the subset of miscellaneous compounds, it was observed that compounds **21**, **22**, and **24** exhibited no activity against SARS-CoV-2 when tested at concentrations close to their corresponding CC_50_ values. Despite the poor results obtained for compound **21**, the investigation of further benzotriazole derivatives is still worthwhile. Indeed, besides our previous work [44], many others illustrated their antiviral activities [69,70,71,72], and benzotriazole itself has been shown to interact with SARS-CoV-2 proteins [73].

Also, the even modest activity of **23** and **25** still suggests the screening of some other cytisine and arylamino enone derivatives, respectively. Indeed, the activity of **23** may be related to the affinity to the α_4_β_2_ subtype of the nicotinic receptor (Ki = 2.3 µM [48]), and the interaction of the SARS-CoV-2 spike protein with the nicotinic receptor is increasingly recognized [74,75]. Compound **25** has been shown to act favorably against prostate and triple-negative breast cancer [76,77], probably via TRAF6 inhibition.

Finally, to confirm the results obtained through real-time PCR, the two most active compounds with the highest SI, along with two compounds from the benzimidazole derivative group, were tested using the plaque assay, a method that assesses the actual number of infectious viral particles present in the samples. The compounds **10**, **11**, and chloroquine were tested at concentrations of 50–25-16.7–5.6 and 1.9 µM, while the benzimidazole derivatives were tested at concentrations of 40–20-6.7–2.2–0.7 µM. The results (Table 2), expressed as the mean number of plaques (PFU/mL) and as the percentage of viral replication (in brackets), confirm that all tested compounds can inhibit viral replication and infectivity in a dose-dependent manner.

## 3. Materials and Methods

### 3.1. Chemistry

#### 3.1.1. General Information

Chemicals and solvents were purchased from Sigma-Aldrich (Milan, Italy). Mps: Büchi apparatus, uncorrected. ^1^H NMR spectra and ^13^C NMR spectra were recorded using a Jeol instrument at 400 and 101 MHz, respectively; chemical shifts are reported as δ (ppm) and are referenced to the solvent signal: DMSO-d6, quintet at 2.5 ppm (^1^H), septet at 39.5 ppm (^13^C); *J* in Hz. Elemental analyses were performed on a Flash 2000 CHNS (Thermo Scientific, Waltham, MA, USA) instrument in the Microanalysis Laboratory of the Department of Pharmacy, University of Genova. Results of elemental analyses indicated that the purity of all compounds was ≥95%. Benz: benzimidazole ring; Q: quinolizidine ring.

#### 3.1.2. General Procedure for the Preparation of Thiosemicarbazones

To a solution of the proper 5-acetyl benzimidazole (0.80 mmol) [43,59] in ethanol (2 mL), a solution of thiosemicarbazide (0.85 mmol) in water (2.8 mL) and glacial acetic acid (0.22 mL) was added. The mixture was refluxed for 3 h under stirring. The reaction mixture was then evaporated to dryness, affording an oily residue that was purified by CC (SiO_2_, CH_2_Cl_2_+2% DEA), affording the final product as a white solid.

2-(1-{2-Benzyl-1-[((1S,9aR)-octahydro-2H-quinolizin-1-yl)methyl]-1H-benzo[d]imidazol-5-yl}-ethylidene)hydrazine-1-carbothioamide (**14**) Yield: 78%; m.p. 205–207 °C. ^1^H NMR (400 MHz, DMSO-D_6_) δ 10.16 (s, 1H, NH), 8.21 (s, 1H, NH_2_), 8.11 (d, *J* = 1.7 Hz, 1H, Benz.), 7.93 (s, 1H, NH_2_), 7.90 (dd, *J* = 8.6, 1.8 Hz, 1H, Benz.), 7.40 (d, *J* = 8.7 Hz, 1H, Benz.), 7.37–7.20 (m, 5H, Ar), 4.35–4.23 (m, 2H, CH_2_-Q and 4.28, s, 2H, CH_2_-Ar), 2.82–2.75 (m, 2H, Hα N of Q), 2.35 (s, 3H, CH_3_), 2.12–2.07 (m, 1H, Q), 1.99 (s, 1H, Q), 1.93–1.80 (m, 3H, Q), 1.76–1.68 (m, 1H, Q), 1.60–1.50 (m, 3H, Q), 1.45–1.30 (m, 2H, Q), 1.27–1.11 (m, 3H, Q). ^13^C NMR (101 MHz, DMSO-D_6_) δ 178.63, 154.52, 148.85, 142.25, 136.76, 136.55, 131.37, 128.77 (2C), 128.50 (2C), 126.64, 120.88, 117.57, 109.82, 64.11, 56.72, 56.54, 41.60, 38.17, 33.30, 28.8–5, 25.60, 25.09, 24.60, 20.61, 14.35. Anal. calcd. for C_27_H_34_N_6_S: % C 68.32, H 7.22, N 17.71, S 6.75; found: % C 67.98, H 7.18, N 17.96, S 6.87.

2-(1-{2-[(1H-Benzo[d][1,2,3]triazol-1-yl)methyl]-1-[3-(N,N-diethylamino)propyl]-1H-benzo-[d]imidazol-5-yl}ethylidene)hydrazine-1-carbothioamide (**15**) Yield: 64%; m.p. 195–197 °C. ^1^H NMR (400 MHz, DMSO-D_6_) δ 10.14 (s, 1H, NH), 8.21 (s, 1H, NH_2_), 8.12 (d, *J* = 1.7 Hz, 1 arom. H), 8.08 (d, *J* = 8.3 Hz, 1 arom. H), 7.98 (dd, *J* = 8.7, 1.8 Hz, 2H, 1 arom. H + 1 NH_2_), 7.84 (d, *J* = 8.3 Hz, 1 arom. H), 7.60–7.50 (m, 2 arom. H.), 7.46–7.38 (m, 1 arom. H), 6.44 (s, 2H, CH_2_-Ar), 4.38 (t, *J* = 7.3 Hz, 2H,CH_2_CH_2_CH_2_N(Et)_2_), 2.39 (q, *J* = 7.1 Hz, 4H, N(CH_2_CH_3_)_2_), 2.32 (s, 3H, CH_3_), 2.28 (t, *J* = 6.8 Hz, 2H, CH_2_CH_2_CH_2_N(Et)_2_), 1.72 (p, *J* = 7.0 Hz, 2H, CH_2_CH_2_CH_2_N(Et)_2_), 0.89 (t, *J* = 7.1 Hz, 6H, N(CH_2_CH_3_)_2_). ^13^C NMR (101 MHz, DMSO-D_6_) δ 178.65, 149.23, 148.61, 145.34, 141.92, 135.98, 133.24, 131.97, 127.63, 124.20, 121.77, 119.28, 118.09, 111.01, 110.22, 48.94, 45.84 (2C), 44.56, 41.70, 26.53, 14.29, 11.38 (2C). Anal. calcd. for C_24_H_31_N_9_S: % C 60.35, H 6.54, N 26.39, S 6.71; found % C 60.48, H 6.34, N 26.31, S 6.58.

### 3.2. Cytotoxicity Assay

Vero E6 cells (Monkey Kidney Epithelial Cells, ATCC C1008) were maintained in DMEM medium (EuroClone, Milan, Italy) supplemented with 10% heat-inactivated fetal calf serum (EuroClone, Milan, Italy), 2 mM glutamine (EuroClone, Milan, Italy), 100 units/mL of penicillin, and 100 μg/mL of streptomycin (EuroClone, Milan, Italy). For the cytotoxicity assay, Vero E6 cells were seeded into 96-well plates at concentration of 1 × 10^4^ cells/well. After 24 h of incubation, the cells were treated with serial 2-fold dilutions of compounds, in a final volume of 200 μL, in duplicate. After incubation for 72 h at 37 °C in 5% CO_2_, cell viability was measured by 3-(4,5-dimethylthiazol-2-yl)-2,5-diphenyltetrazolium (MTT) (Merk, Darmstadt, Germany) assay, as previously described [78]. Percentage of viable cells was calculated using untreated cells as control (100% viability) using the formula [(sample absorbance—cell free sample blank)/mean media control absorbance] × 100. The 50% cytotoxic concentration (CC50) causing 50% reduction in Vero E6 cells’ viability with respect to untreated control cells was determined using Gene5 software. Morphological changes of Vero E6 cells were also observed by light microscopy.

### 3.3. Vero E6 Cells Infection, Treatment and Evaluation of the Antiviral Activity

SARS-CoV-2 belonging to the B1.1 lineage was isolated from the nasal-pharyngeal swab of an Italian patient, as described [79]. Vero E6 cells were seeded into 96-well plates at a density of 1.3 × 10^4^ cells/well and were incubated for 24 h at 37 °C, 5% CO_2_. Cells were infected with an MOI of 0.05 (1000 PFU/well/30 µL) and incubated for 2 h at 37 °C, 5% CO_2_. After removal of virus inoculum, cells were treated with the compounds (dose range 2.2–60 µM; 200 µL/well final volume) and incubated for 72 h at 37 °C, 5% CO_2_. Chloroquine was used as the control drug.

SARS-CoV-2 replication was evaluated by RNA isolation from cells’ supernatants followed by specific qRT-PCR, targeting the N1 gene [80,81]. SARS-CoV-2 viral load data (copies/µL) were normalized versus untreated infected controls according to the following formula: % SARS-CoV-2 replication = 100 × (viral load treated sample/viral load untreated control). Data were plotted as a function of drug concentration and curve fitting was obtained by non-linear regression analysis using a four-parameter logistic method (software GraphPad Prism v. 6.0, GraphPad, La Jolla, CA, USA). The IC_50_ value was extrapolated as the concentration that induced a 50% inhibition of viral replication.

Antiviral activity of the most active compounds and of the reference drug chloroquine was also evaluated by plaque assay. Vero E6 cells were seeded in 6-well plates (400,000 cells/well) for 24 h at 37 °C. Virus inoculum (50 PFU/well) was added to the wells for 2 h at 37 °C. Subsequently, virus inoculum was removed, and cells were covered with 0.3% agarose dissolved in cell medium in the presence or not of different concentrations of compounds (range 0.7 µM–50 µM) at 37 °C for 72 h. Cells were fixed with 4% formaldehyde solution (Merk, Darmstadt, Germany) and, after agarose removal, stained with methylene blue (Merk, Darmstadt, Germany). Results were expressed as Plaque Forming Unit (PFU)/mL, and as percentage of virus replication, compared to untreated infected cells.

## 4. Conclusions

Many chemical classes of compounds are able to inhibit the in vitro replication of SARS-CoV-2 (the virus responsible for COVID-19 disease), with valuable potencies and various mechanisms of action. However, the current COVID-19 therapy is far from being satisfactory, and the search for novel, more effective compounds continues to be a priority.

Thus, we deemed it worthwhile to test, against SARS-CoV-2 replication, several compounds previously identified by our group as endowed with potent antiviral and/or anti-parasitic activity, and which are structurally related to some privileged scaffolds discussed in the Introduction (riminophenazines, 4-aminoquinolines, benzimidazole derivatives, etc.), and in most cases characterized by the presence of the bulky quinolizidine and pyrrolizidine rings.

Indeed, many of the twenty-five tested compounds (**2**, **3**, **10**, **11**, **13**–**15**, and **18**–**20**) displayed valuable antiviral activity (IC_50_ in the range 1.5–28 µM), and, in relation to their cytotoxicity versus Vero E6 cells, only six of them (**10**, **11**, **13**–**15**, and **19**) exhibited a promising selectivity index (SI). Evidence in the literature indicates that drugs and compounds tested against SARS-CoV-2 and related coronaviruses display a variable SI, which can be higher or lower, depending on the sensitivity of the animal or human cell line used as host cells for viral replication [82,83]. Therefore, the present compounds require a more in-depth analysis to better define their antiviral efficacy.

In particular, the bulkier and lipophilic compounds **10** and **11** were found to be more potent than the reference drug chloroquine, even having a doubled SI value (20 versus 11). These compounds have been shown to exhibit favorable preclinical pharmacological profiles [40,41], and therefore other chloroquine analogs bearing an even bulkier basic side chain will be investigated.

On the other hand, the benzimidazole derivatives **13**–**15** and **18**–**20** warrant further investigation of some analogs to, potentially, furnish improved antiviral agents. Similarly, despite the modest activity, compounds **23** and **25** also deserve further consideration as model compounds, given the recently shown antiviral activity of some cytisine (nicotinic ligand) analogs [49,74] and the promising anti-TRAF6 and anticancer activities of **25**, which are currently under investigation by some of the authors.

The definition of the mechanism of action of the diverse chemotypes identified will be investigated as a further extension of this exploratory work. Moreover, due to the mutational landscape of SARS-CoV-2 that impacts its transmissibility and antigenicity, the most promising compounds will be then tested against different SARS-CoV-2 variants with a view to probing their potential broad-spectrum antiviral activity and mutation-induced drug resistance leading to targeting failure.

## Figures and Tables

**Table 1:** Cytotoxicity (CC_50_), antiviral activity (IC_50_), and SI of compounds **1**–**25** and CQ.

Compound	Vero E6 CC_50_ (µM) ^a^	SARS-CoV-2 IC_50_ (µM) ^a^	S.I.
**1**	3.14	>2	
**2**	2.81	1.5	1.9
**3**	10.5	4.0	2.6
**4**	2.19	>1	
**5**	3.20	>2	
**6**	5.58	>3	
**7**	2.62	(20.5%) ^b^	
**8**	1.74	NA	
**9**	1.88	NA	
**10**	44.0	2.2	20
**11**	74.5	3.7	20.1
**12**	>100	>100	
**13**	96.1	20.0	4.8
**14**	70.0	12.2	5.7
**15**	67.1	14.9	4.5
**16**	37.7	>20	
**17**	191.9	>60	
**18**	107.4	28.1	3.8
**19**	76.3	15.3	5.0
**20**	19.4	6.95	2.8
**21**	25.4	>20	
**22**	26.8	>20	
**23**	>100	95.6	>1.1
**24**	32.9	>10	
**25**	>100	90.1	>1.1
**CQ**	96.3	8.50	11.3

^a^ The results are the means of three different experiments performed in duplicate, SD < 15%; ^b^ % inhibition of viral replication at 1 μM conc.; NA = not active at 1 μM conc.

**Table 2:** Virucidal activity ^a^ of tested compounds and CQ.

Cpd	Mean PFU/mL (% Replication) at the Indicated μM Concentrations										
	Untreated Infected Cells	0.7	1.9	2.2	5.6	6.7	16.7	20	25	40	50
**10**	102.5 (100)	-	83.5 (84.5)	-	42.0 (40.9)	-	10.0 (9.8)	-	0.0 (0)	-	-
**11**	63.0 (100)	-	61.0 (96.8)	-	26.3 (41.8)	-	3.0 (4.8)	-	-	-	0.0 (0)
**CQ**	85.0 (100)	-	73.3 (86.2)	-	54.3 (63.8)	-	0.0 (0)	-	-	-	0.80 (0)
**18**	44.5 (100)	-	-	28 (69.9)	-	20 (44.9)	-	13 (29.2)	-	1 (2.2)	-
**20**	53 (100)	67 (>100)	-	57 (>100)	-	34 (63)	-	3 (5)	-	-	-

^a^ The results are the means of three independent experiments performed in duplicate, SD < 15%. The percentage of virus infectivity compared to the untreated control is indicated in brackets.

## Data Availability

Data are contained within the article.

## Supplementary Materials

The following supporting information can be downloaded at: https://www.mdpi.com/article/10.3390/ph17121668/s1, ^1^H and ^13^C NMR spectra of the newly synthesized compounds **14** and **15**.
