# Supplementary material for: In Vitro Screening of an In-House Library of Structurally Distinct Chemotypes Towards the Identification of Novel SARS-CoV-2 Inhibitors
Source: Pharmaceuticals (Basel). 2024 Dec 11;17(12):1668. doi: 10.3390/ph17121668 (PMC11676875; doi:10.3390/ph17121668)

In vitro screening of an in-house library of structurally distinct chemotypes towards the identification of novel SARS-CoV-2 inhibitors

Michele Tonelli [1,*], Anna Sparatore [2,*], Ivan Bassanini [3], Valeria Francesconi [1], Fabio Sparatore [1], Kevin K. Maina [4], Serena Delbue [4], Sarah D'Alessandro [5], Silvia Parapini [6] and Nicoletta Basilico [4,*]

^{1}H NMR of Compound 14 + Aromatic region expansion

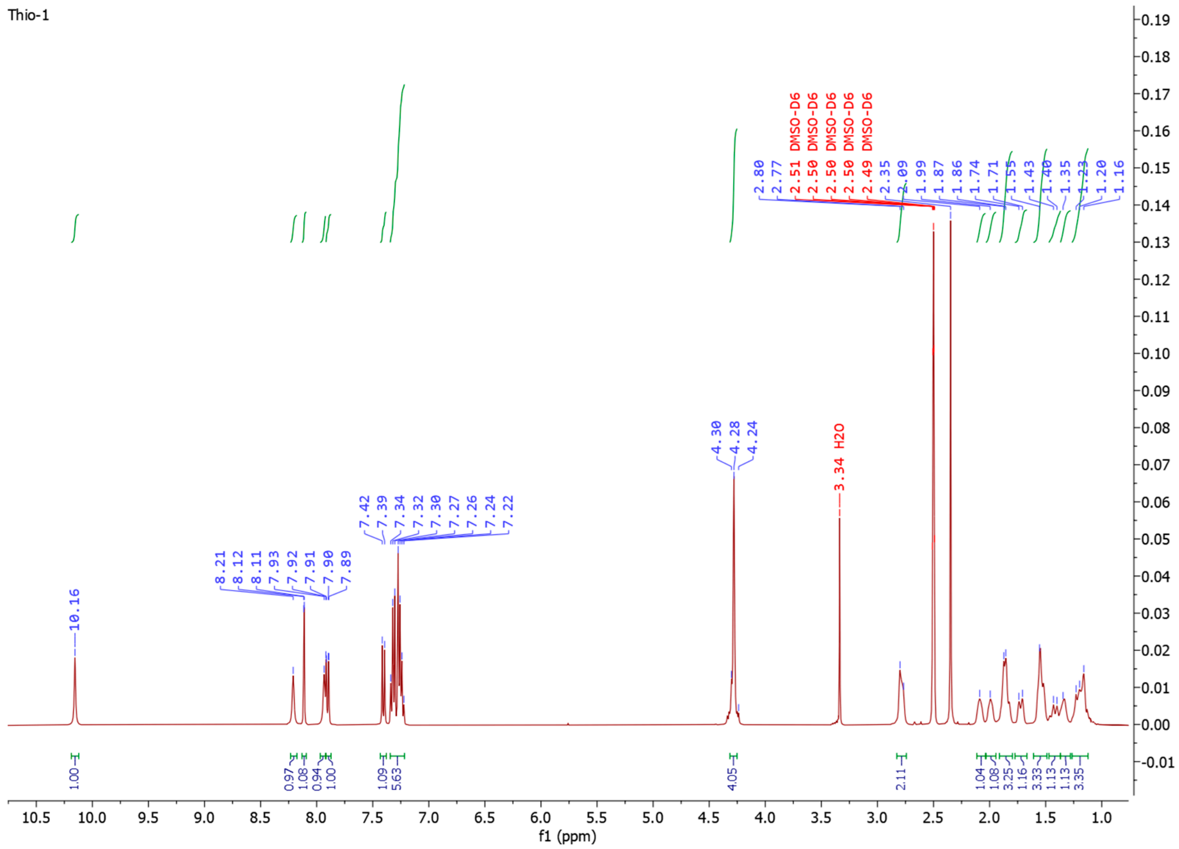

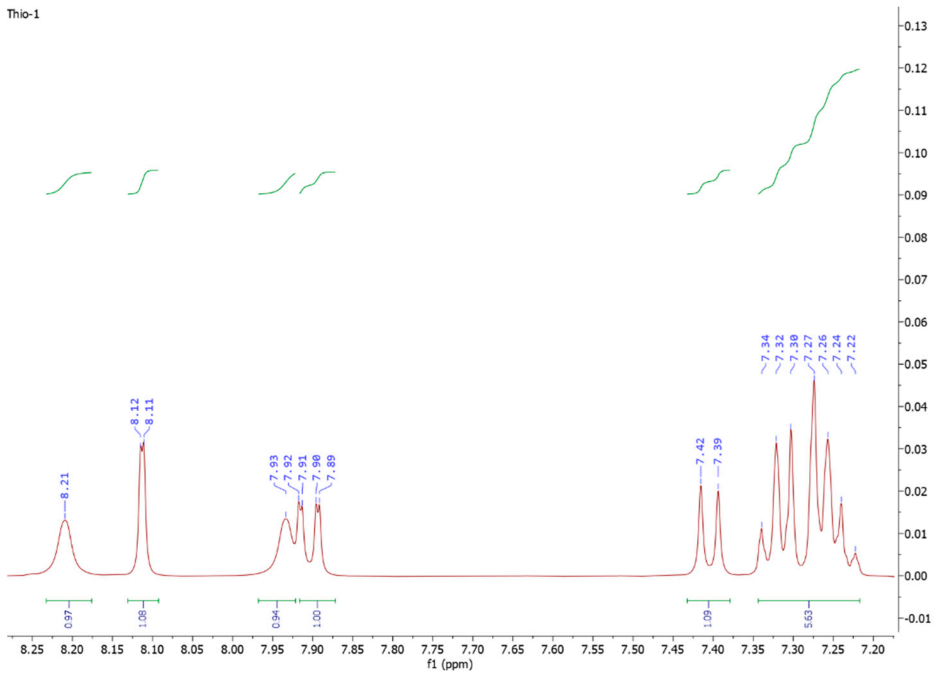

^{13}C NMR of Compound 14

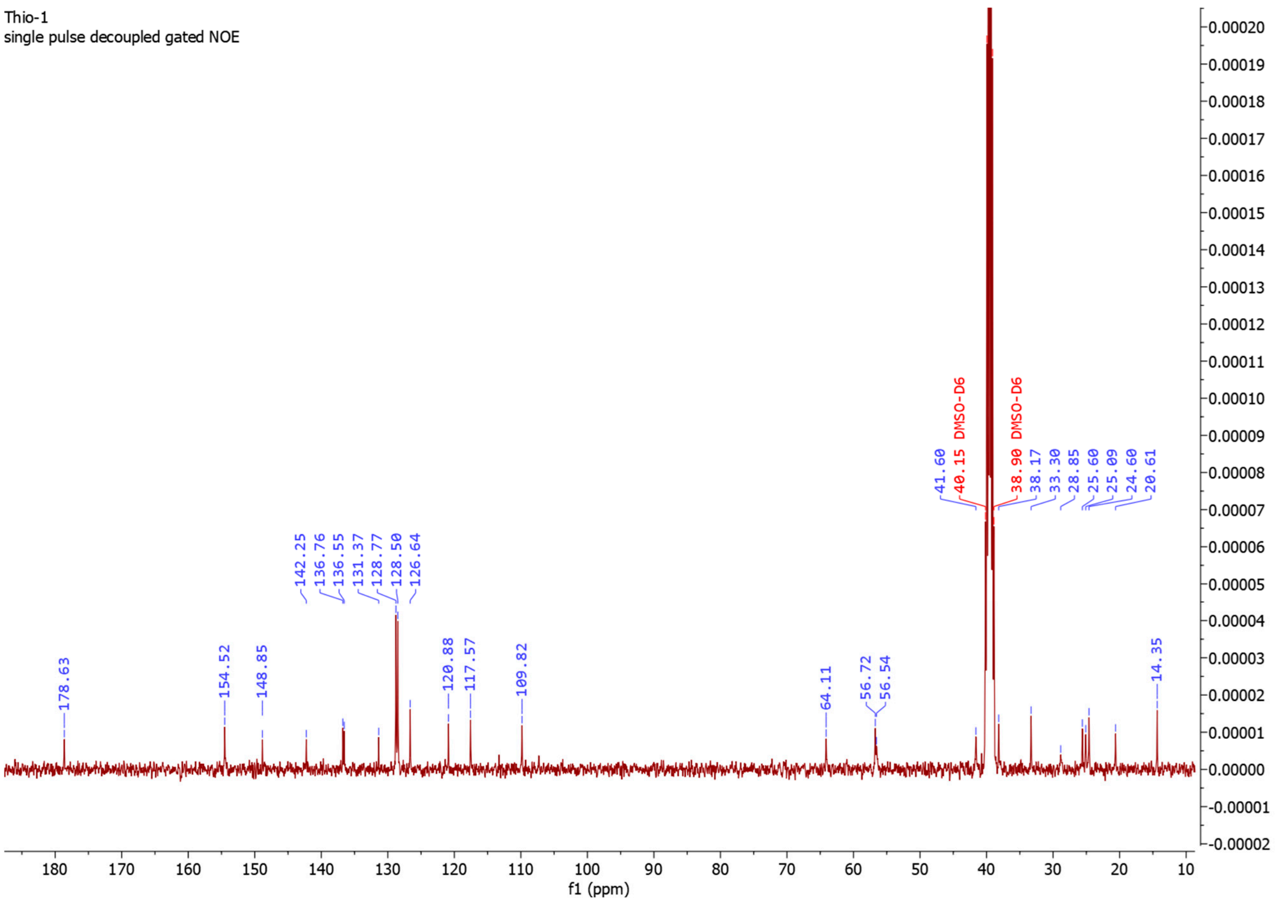

^{1}H NMR of Compound 15 + Aromatic region expansion

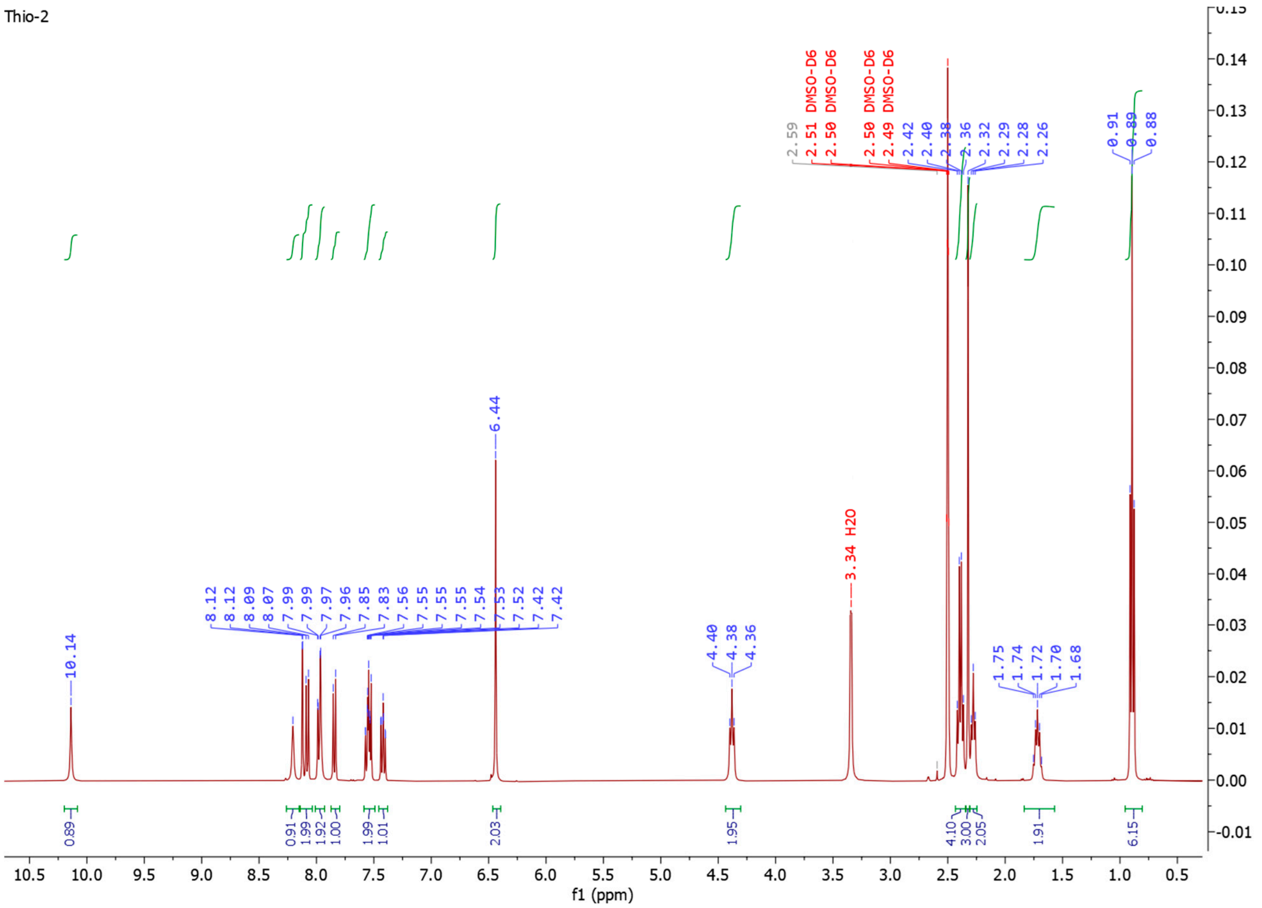

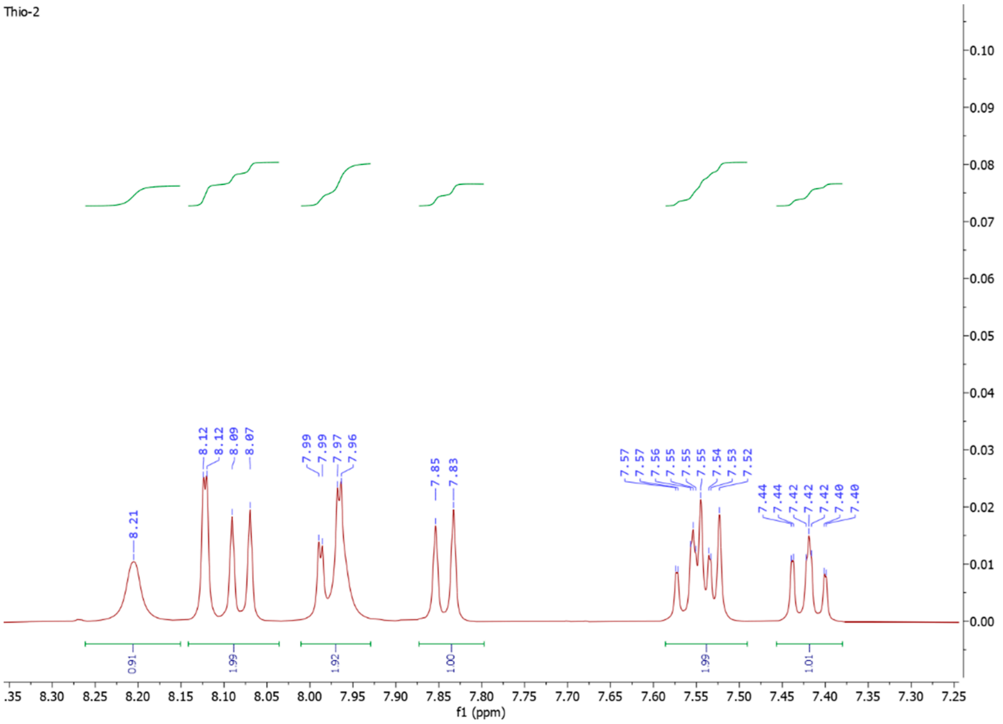

^{13}C NMR of Compound 15

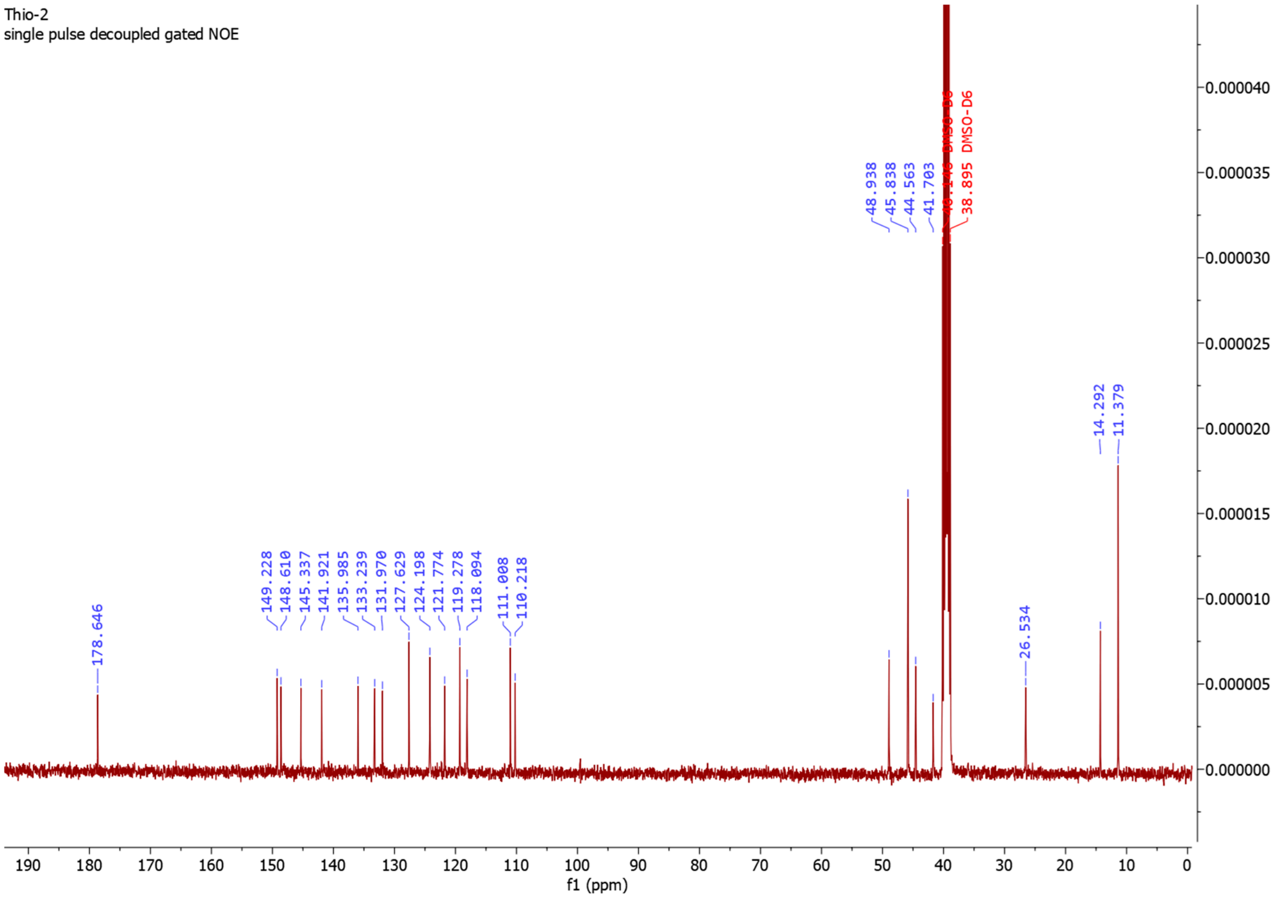

Supplement: Supplementary file 1 [file pharmaceuticals-17-01668-s001.zip › pharmaceuticals-3331200-supplementary.pdf]
